# The relationship between lower urinary system symptoms and the level of independence and quality of life in children with Duchenne muscular dystrophy

**DOI:** 10.1007/s00467-024-06419-0

**Published:** 2024-06-01

**Authors:** Demet Öztürk, Aynur Ayşe Karaduman, Türkan Akbayrak

**Affiliations:** 1 Department of Physiotherapy and Rehabilitation, Faculty of Health Sciences, Lokman Hekim University, 06530 Ankara, Turkey; 2 Faculty of Physical Therapy and Rehabilitation, Hacettepe University, Ankara, Turkey

**Keywords:** Duchenne muscular dystrophy, Lower urinary tract symptoms, Activities of daily living, Quality of life, Child, Enuresis

## Abstract

**Background:**

The purpose was to investigate the frequency of lower urinary tract symptoms (LUTS) and lower urinary tract dysfunction (LUTD) in Duchenne muscular dystrophy (DMD) and the relationship between these symptoms and independence and quality of life (QoL).

**Methods:**

The cross-sectional study included children aged 5–18 years and diagnosed with DMD and their families. Data were collected using the Dysfunctional Voiding and Incontinence Scoring System (DVISS), the Barthel Index, and the Pediatric Quality of Life™ 3.0 Neuromuscular Module (PedsQL-NMM).

**Results:**

The study was completed with 45 children with DMD. LUTS was found in 86.66% and LUTD was found in 44.44%. The most common symptom was holding maneuvers (62.22%). Other common symptoms were urinary urgency (55.55%), daytime urinary incontinence (46.66%), and enuresis (31.11%). There was a significant correlation of the DVISS with the level of independence and QoL (*p* < 0.05). Moreover, higher LUTS score was associated with lower Barthel and PedsQL-NMM scores.

**Conclusion:**

LUTS is a neglected condition, although it is frequently seen in children with DMD.

Clinical trial registration: NCT05464446

**Graphical Abstract:**

**Figure:**
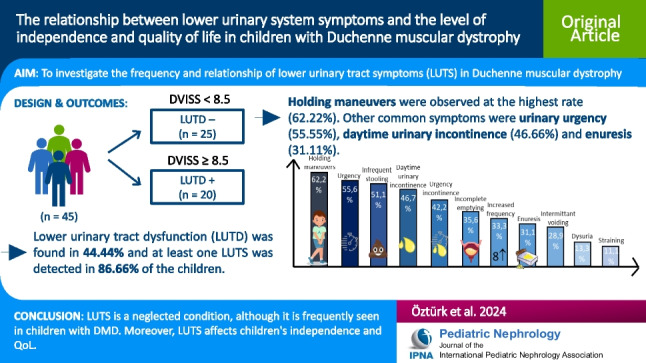
A higher resolution version of the Graphical abstract is available as Supplementary information

**Supplementary Information:**

The online version contains supplementary material available at 10.1007/s00467-024-06419-0.

## Introduction

Duchenne muscular dystrophy (DMD) is an X-linked recessive genetic disorder that occurs once every 5000 live male births [1, 2]. It is a type of neuromuscular disease caused by a mutation in the dystrophin gene [3]. Dystrophin is a protein complex that plays an important role in the continuity of muscle function in the body. Dystrophin deficiency in children with DMD also causes dysfunction of smooth muscle cells in the bladder and urethra [4]. Lower urinary tract symptoms (LUTS) are common in children with DMD due to smooth muscle dysfunction in the urinary tract [5].

In the literature, the presence of LUTS has been reported in approximately half of individuals with DMD [4]. However, the prevalence of LUTS varies because studies have included muscular dystrophies other than DMD, examined broad age groups, and used different questionnaires. More information is needed on the frequency and distribution of LUTS. In addition, it is important to examine the relationship between LUTS and related factors.

The primary objective of this study was to determine the frequency and distribution of LUTS in children with DMD. Second, the study aimed to examine the relationship between LUTS and children's participation in activities of daily living (ADL) and their quality of life (QoL).

## Materials and methods

This cross-sectional study was approved by the Non-Interventional Clinical Research Ethics Committee of Hacettepe University (GO 21/982). This study was registered with clinical trials (NCT05464446) and conducted in accordance with the Declaration of Helsinki. All data were recorded after obtaining the verbal and written consent from the parents and children who met the inclusion criteria.

### Participants

**Fig. 1:**
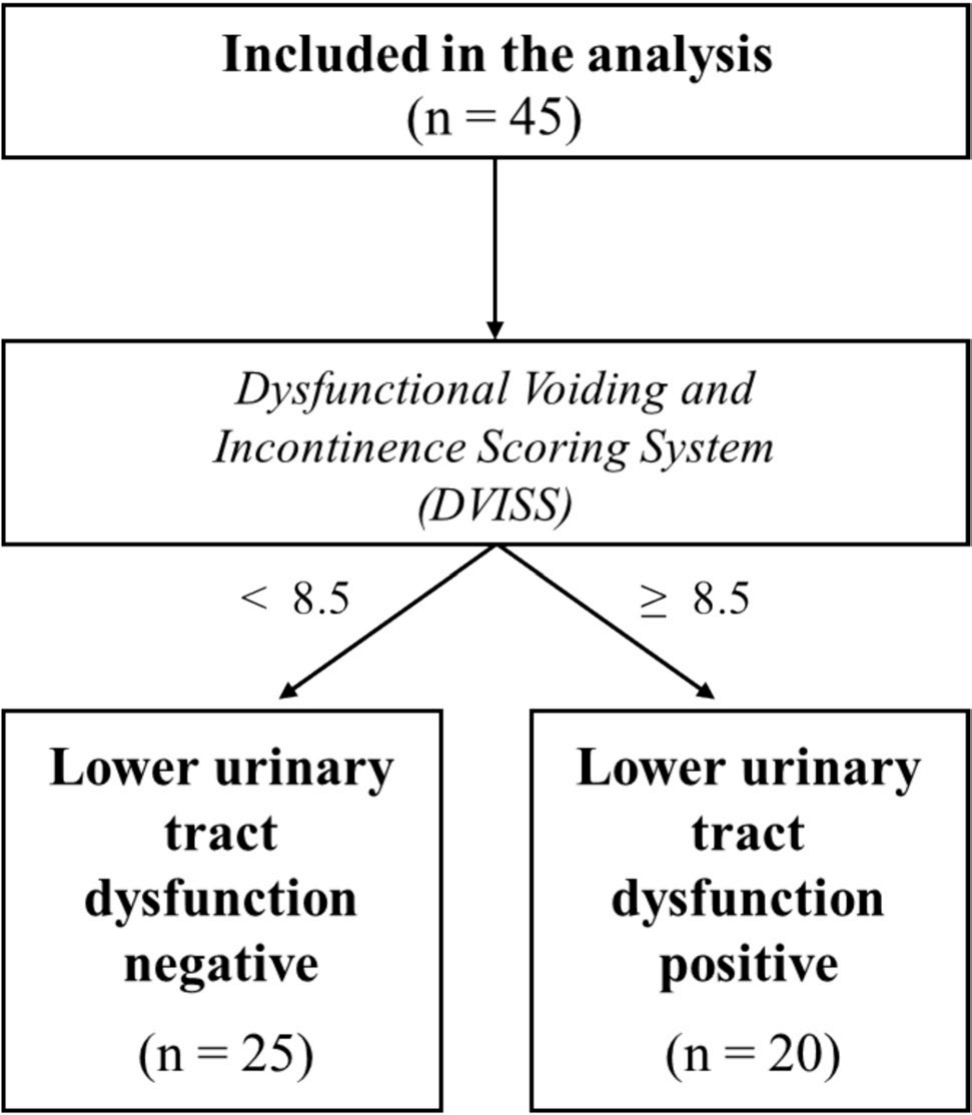
Study flowchart

The study consisted of children aged 5–18 years with a diagnosis of DMD and their families between October 2021 and May 2022. Children with a diagnosis in addition to DMD, children with Becker muscular dystrophy (BMD) and other neuromuscular diseases, and those with cooperation problems and difficulty in understanding the Turkish language were excluded from the study. Of the 86 patients who presented to the Neuromuscular Diseases Application and Research Center, 41 patients who did not meet the inclusion criteria were excluded from the study. As a result, the study was completed with 45 patients with DMD and their families (Fig. 1).

### Measures

The children’s demographic characteristics and disease information were obtained using a general assessment form. The general assessment form asked about the child's age, body weight, height, age at diagnosis, age of toilet training, ambulation level, steroid use, and urological control. The participants then completed validated DMD-specific questionnaires.

The functional levels of the upper and lower extremities of children with DMD were assessed using the Brooke Upper Extremity Functional Rating Scale (BUEFRS) and the Vignos Scale. The BUEFRS consists of 6 stages. Each stage is divided into early, intermediate, and late stages [6]. The Vignos Scale consists of 10 stages [7]. The first three stages were classified as “early stage”, stages of four and five as “intermediate stage”, and stages six through ten as “late stage” [8]. Individuals with DMD were then assessed for LUTS, ADL and QoL.

The Dysfunctional Voiding and Incontinence Scoring System (DVISS) was used to evaluate the presence and frequency of LUTS in children. The scale assesses daytime symptoms, nighttime symptoms, voiding habits, bowel habits, and QoL of children with DMD [9]. LUTS were subjectively assessed using the DVISS. The International Children's Continence Society (ICCS) recommends the DVISS for the evaluation of the function of the lower urinary tract function [10]. The total score of the scale ranges from 0 to 35, and the increase in the total score indicates an increase in the severity of LUTS. The QoL questionnaire score is not added to the total score, only the symptom scores are added to obtain the total score of the questionnaire. Akbal et al. showed that a score of 8.5 may be an optimal threshold score to determine whether the child has clinically significant lower urinary tract dysfunction (LUTD), with a sensitivity of 90% and a specificity of 90% [9]. In our study, we used this cut-off value to determine the presence of LUTD.

The Barthel Index was used to measure children's participation in ADL and their level of independence in these activities. The scale evaluates feeding, bathing, self-care, dressing, grooming, bowel and bladder control, toilet use, transfer activities, ambulating and stair climbing activities. The scale has 10 questions and the total score ranges from 0 (completely dependent) to 100 (independent). Higher scores indicate greater independence in ADL [11, 12].

The Pediatric Quality of Life ™ 3.0 Neuromuscular Module (PedsQL-NMM) was used to evaluate QoL of children with DMD. The PedsQL-NMM was developed by Varni et al. to evaluate QoL in neuromuscular disorders such as DMD [13]. Therefore, the PedsQL-NMM is valid and reliable in DMD. This module, which has 25 items, consists of three subscales: About My/My Child’s Neuromuscular Disease (17 items), Communication (3 items), and About Our Family Resources (5 items). The total score ranges from 0 and 100, and as the total score increases, children’s QoL increases [13, 14].

### Statistical analysis

All analyses were performed with SPSS version 26.0. Excel 2016 was used for the frequency distribution graph of the symptoms. Spearman’s correlation test was used to determine the correlation between LUTS and the Barthel Index total score and PedsQL-NMM total score and sub-group scores. A correlation coefficient ≥ 0.80 was considered very strong, 0.60 – 0.79 as strong, 0.40 – 0.59 as moderate, 0.20 – 0.39 as poor, and < 0.20 as very poor. A p-value of less than 0.05 was considered a statistically significant level [15]. The presence of LUTD in children was determined using the cut-off value in the DVISS score. The Chi-square test was used to examine the difference between subjects with and without LUTD in terms of Barthel Index subscales and urologist control categories.

## Results

Children aged 5–18 years were included in this study, and the study was completed with 45 children with DMD (age: 9.00 ± 3.32 years). Mean body mass index was 18.83 ± 2.81 kg/cm^2^ and mean age at toilet training was 2.87 ± 0.87 years. Of the 45 children included in the study, 38 were in the ambulatory stage and 7 were in the non-ambulatory stage. In addition, the levels of upper and lower extremity functionality were high. Patient characteristics related to the children’s demographics and disease are shown in Table 1.

**Table 1:** Characteristics of children with DMD

Demographic characteristics		Total (*n* = 45)
		Median (25–75. Percentile)
Age (years)		8.00 (6.00–11.00)
Body weight (kg)		28.00 (20.00–37.00)
Height (cm)		121.00 (110.00–136.00)
BMI (kg/m^2^)		17.84 (16.97–21.40)
Diagnosis age (years)		3 (1.50–6.00)
Toilet training age (years)		2.50 (2.50–3.00)
Time of non-ambulatory (years) (min–max)		0.71 ± 1.57 (0.00 – 6.00)
Functional level and medical characteristics		*n* (%)
BUEFRS	Early stage	40 (88.89)
	Middle stage	3 (6.67)
	Late stage	2 (4.44)
Vignos Scale	Early stage	36 (80.00)
	Middle stage	1 (2.22)
	Late stage	8 (17.78)
Using steroid	Yes	36 (80.00)
	No	6 (13.33)
	Quit	3 (6.67)
Ambulation	Ambulatory	38 (84.44)
	Non-ambulatory	7 (15.56)

*SD* Standard deviation; *BMI* Body mass index; *BUEFRS* Brooke Upper Extremity Functional Rating Scale

### Lower urinary tract symptoms and dysfunction

Twenty children with a DVISS score of 8.5 and above were included in the LUTD-positive group, and 25 children with a score below 8.5 were included in the LUTD-negative group. LUTD was found in 20 (44.44%) of the children.

At least one lower urinary tract symptom was detected in 86.66% (*n* = 39) of the children. Five children (11.11%) had one lower urinary tract symptom, 13 children (28.88%) had two to four LUTS, and 21 children (46.66%) had more than five LUTS (n_5 symptoms_ = 6; n_6 symptoms_ = 4; n_7 symptoms_ = 5; n_8 symptoms_ = 3; n_9 symptoms_ = 3).

**Fig. 2:**
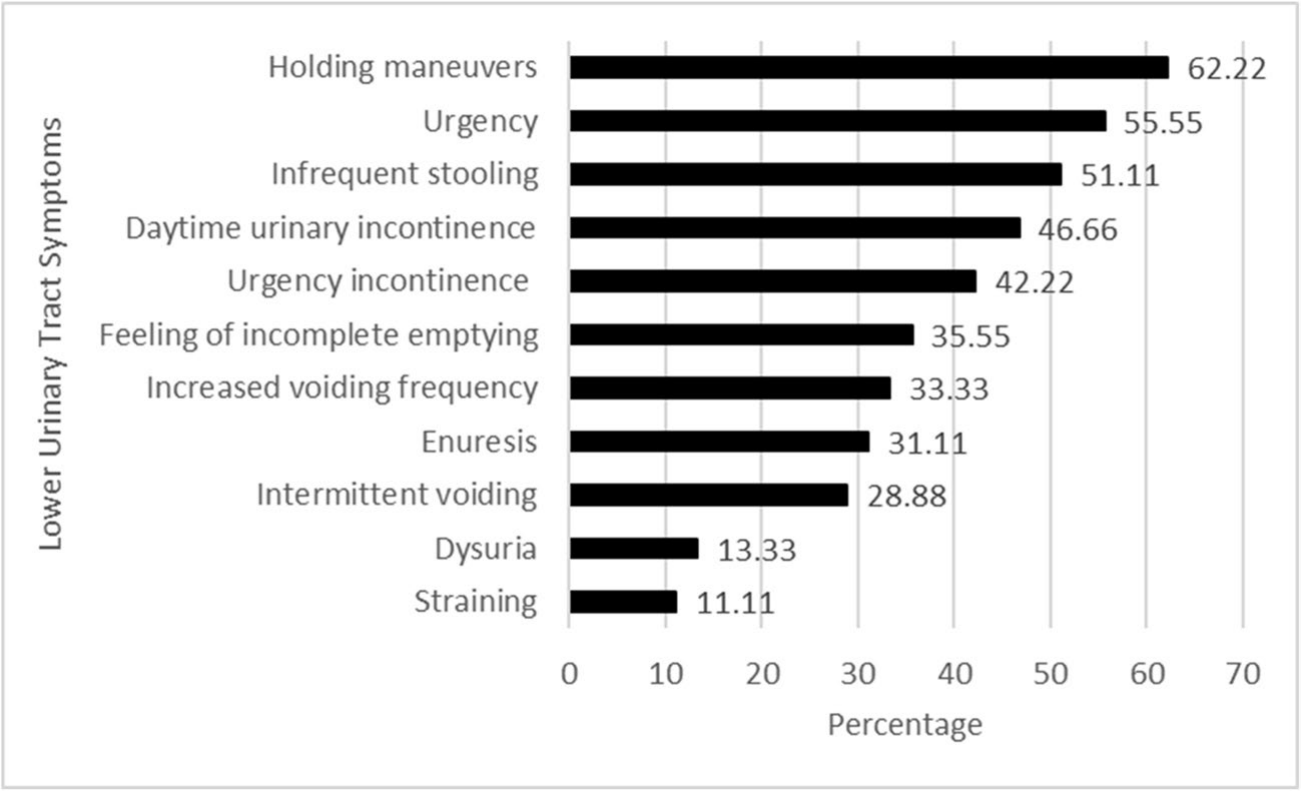
Frequency of lower urinary tract symptoms in children with DMD

For children with DMD, the most prevalent symptoms were holding maneuvers (62.22%) and urgency (55.55%). Daytime urinary incontinence was found in 21 children (46.66%), and enuresis (nighttime/nocturnal incontinence) was found in 14 children (31.11%). Urgency incontinence was found in 19 children (42.22%), feeling of incompletely emptying the bladder in 16 children (35.55%), increased voiding frequency in 15 children (33.33%), and intermittent voiding in 13 children (28.88%). In addition, dysuria (burning or discomfort during micturition) was found in 6 children (13.33%) and straining (intense effort to increase intra-abdominal pressure to void) in 5 children (11.11%). In addition, 23 children (51.11%) had infrequent stooling. The frequency of LUTS in children with DMD is shown in Fig. 2.

It was also found that 36 of 45 children with DMD who participated in the study had never been to a urologist. It was determined that 80.00% of the children with DMD, 84.00% of LUTD-negative children, and 75.00% of LUTD-positive children, had never been to a urologist in their lives (Table 2).

**Table 2:** Urologist follow-up information of children with DMD

Variable		LUTD positive *n* (%)	LUTD negative *n* (%)	χ2	*p*
		20 (44.44)	25 (55.56)		
Urologist follow-up	None	15 (75.00)	21 (84.00)	0.563	0.482
	Yes	5 (25.00)	4 (16.00)		

Chi-square test; *p* < 0.05

*LUTD* Lower Urinary Tract Dysfunction

### Participation in activities of daily living

Independence in ADL was significantly lower in the LUTD-positive group (*p* < 0.05). It was shown that children with DMD in the LUTD-positive group were more dependent on ADL. There was also a moderately significant negative correlation between the level of independence in ADL and the DVISS total score (r = -0.467; *p* = 0.001). As a result, a significant association was found between higher LUTS total score and lower Barthel total score. Data on the participation in ADL of children with DMD are presented in Table 3.

**Table 3:** Participation in activities of daily living of children with DMD

Total score	DVISS Total		LUTD positive (*n* = 20)	LUTD negative (*n* = 25)	Z	*p*
	r	*p*	Mean ± SD			
Barthel Index total	**-0.467**	0.001*^b^	71.75 ± 20.60	86.20 ± 22.51	-3.346	0.001*^a^
Subgroups			*n* (%)		χ2	*p*
Barthel Index categorical	Independent (100)		0 (0.00)	8 (32.00)	16.108	**0.003**^b^*
	Mildly dependent (91–99)		2 (10.00)	8 (32.00)		
	Moderately dependent (62–90)		13 (65.00)	6 (24.00)		
	Highly dependent (21–61)		5 (25.00)	2 (8.00)		
	Completely dependent (0–20)		0 (0.00)	1 (4.00)		

^a^Mann-Whitney U test, ^b^Chi-square test

*DVISS* Dysfunctional Voiding and Incontinence Scoring System; *LUTD* Lower Urinary Tract Dysfunction; *SD* Standard deviation; *p < 0.05

### Quality of life

There were differences between the groups in the communication sub-dimension (parent-reported), the disease/symptomatology sub-dimension (child-reported), and the PedsQL-NMM total score (child-reported) (*p* < 0.05). QoL was significantly lower in the LUTD-positive group for these parameters. Moreover, there was a weak negative correlation between the disease/symptomatology sub-dimension (child-reported) and the PedsQL-NMM total score (child-reported) and the DVISS total score (*r* = -0.363, *p* = 0.014; *r* = -0.378, *p* = 0.010) (Table 4).

**Table 4:** Quality of life of children with DMD

Quality of life	LUTD positive (*n* = 20)	LUTD negative (*n* = 25)	Z	*p*	DVISS Total	
	Median (25–75. Percentile)	Median (25–75. Percentile)			r	*p*
Parent						
PedsQL NMM—Disease/symptomatology	70.58 (58.09–77.94)	76.47 (69.11–83.82)	-1.659	0.097^a^	-0.272	0.071^b^
PedsQL NMM— Communication	62.50 (33.33–79.16)	75.00 (58.33–100.00)	-2.066	0.039*^a^	-0.230	0.128^b^
PedsQL NMM—Family Resources	70.00 (57.50–90.00)	75.00 (60.00–85.00)	-0.230	0.818^a^	-0.022	0.885^b^
PedsQL NMM—Total	65.00 (57.00–80.50)	75.00 (70.00–84.00)	-1.623	0.105^a^	-0.247	0.102^b^
Child						
PedsQL NMM—Disease/symptomatology	80.15 (70.58–83.82)	85.29 (80.88–88.23)	-2.306	0.021*^a^	**-0.363**	0.014*^b^
PedsQL NMM—Communication	33.33 (0.00–95.83)	75.00 (0.00–100.00)	-0.436	0.663^a^	-0.109	0.475^b^
PedsQL NMM—Family Resources	62.00 (0.00–75.00)	45.00 (0.00–70.00)	-0.733	0.464^a^	0.039	0.800^b^
PedsQL NMM—Total	78.50 (68.82–84.50)	85.29 (78.00–87.00)	-2.507	0.012*^a^	**-0.378**	0.010*^b^

^a^Mann-Whitney U test, ^b^Spearman Correlation test

*LUTD* Lower Urinary Tract Dysfunction; *DVISS* Dysfunctional Voiding and Incontinence Scoring System; *PedsQL NMM* The Pediatric Quality of Life™ 3.0 Neuromuscular Module; *SD* Standard deviation; *p < 0.05

## Discussion

This study examined the relationship between the severity of LUTS and parameters of ADL and QoL in children with DMD and found LUTS in 86.66% and LUTD in 44.44% of cases. Moreover, higher LUTS scores were associated with lower Barthel and PedsQL-NMM scores. A significant difference was found between the LUTD-positive and LUTD-negative groups in terms of independence in ADL and QoL. These parameters were better in the LUTD-negative group.

In the literature, the prevalence of LUTS in individuals with DMD varies from 50 to 90% due to the use of different measures to assess symptoms. Furthermore, different symptoms have been investigated, a wide age range (childhood to adulthood) has been searched, and BMD has been included in studies [4]. In the cross-sectional study by Bertrand et al. including 56 cases with DMD and/or BMD, 40 individuals (71.42%) reported at least one lower urinary tract symptom [16]. In a study by MacLeod et al., in which 74 individuals with DMD were examined for bladder dysfunction, 46 men with DMD reported LUTS (62.16%) [17]. In the study by van Wijk et al., 170 out of 199 patients with DMD (85.42%) reported one or more LUTS [5]. The study by Lionarons et al. reported that 89.47% of individuals with DMD had at least one bladder and/or bowel symptom [18]. In light of these studies, the presence of one or more LUTS in 86.66% of the cases in our study is consistent with the literature. In addition, our study was age-restricted and included only children with DMD.

In the study by Lionarons et al., the highest rates of symptoms in DMD were found to be enuresis, post-micturition dribble and urinary urgency [18]. Bertrand et al. showed in their retrospective study that the most common symptoms in individuals with DMD and BMD were hesitancy (difficulty in initiating voiding), urinary urgency, and daytime urinary incontinence/enuresis [16]. In our study, the most common symptom was holding maneuvers (62.22%). Other common symptoms were urinary urgency (55.55%), daytime urinary incontinence (46.66%), and enuresis (31.11%). In fact, when daytime urinary incontinence and enuresis are combined under the title of incontinence, it is the most common symptom with a rate of 77.77%. In addition, infrequent stooling was observed in 51.11% of the children with DMD. It has been suggested that the variability in symptoms often seen in the literature may be related to differences in the symptoms evaluated by the researchers and differences in the person (child/family/caregiver) reporting the symptoms. In addition, it was considered that the different functional levels of individuals with DMD included in the studies may have affected the results. In our study, the study sample was limited to childhood, and most of them were in the early stages of the disease. Therefore, it was a homogeneous group consisting of children with a high functional level.

In people with DMD, participation in ADLs gradually decreases as the disease progresses, and dependence on caregivers increases with age [19]. Although studies investigating the relationship between LUTS and ADL in people with DMD are lacking, Lionarons et al. reported that bladder and bowel symptoms would have a negative impact on ADL in 42.00% of individuals with DMD [18]. In our study, an association was found between the severity of LUTS and participation in ADL. The LUTD-negative group had a higher level of participation in ADL and independence. This result was explained by decreasing functionality and increasing dependency. In addition, people with LUTS tend to stay away from activities and prefer social isolation due to shame and social stigma [20]. For these reasons, the existence of an association between LUTS and participation in ADL in children with DMD is consistent.

Due to the fatal nature of DMD, the QoL of individuals with the disease is much lower than that of healthy individuals [21]. For this reason, QoL assessment and mental health screening are recommended at all follow-up visits following guidelines for individuals with DMD [22]. There are many studies in the literature on QoL related to musculoskeletal symptoms in individuals with DMD. Additionally, the relationship between the presence of LUTS and QoL in healthy children is clearly established in the literature [23]. However, few studies have investigated the presence of LUTS in children with DMD. Compared to their healthy peers, children with DMD have developmental and motor delays, lower functional levels, poor posture, and gait disorders. These disease-specific symptoms have been observed to affect the health-related quality of life of people with the condition in a variety of ways [22, 24]. The addition of bladder and bowel symptoms to these symptoms can further exacerbate the negative impact on QoL. In the study by van Wijk et al. in 199 individuals with DMD, the presence of LUTS was shown to reduce QoL [5]. In children with urinary symptoms, emotional well-being and self-esteem are affected. This condition can cause psychological and emotional distress in children, directly impacting their quality of life. Additionally, it can cause other adverse effects such as fatigue, decreased sleep quality, decreased academic performance, decreased participation in sports, and mood disturbances, further affecting overall QoL [23]. In our study, a negative correlation was found between the severity of LUTS and child-reported QoL. In addition, the family-reported communication sub-dimension score, the child-reported disease symptoms sub-dimension score, and the child-reported total QoL score were significantly lower in LUTD-positive children than in LUTD-negative children. The decrease in QoL caused by LUTS was consistent with the literature.

### Strengths

The strengths of our study include the selection of DMD-specific assessment tools (BUEFRS, Vignos Scale, and PedsQL-NMM), the inclusion of a specific disease group, and recruiting a homogeneous group in a specific age group. Moreover, another strength of study is the use of a questionnaire with cut-off value (DVISS) and thus the ability to identify children with LUTD in the clinic without the need for further investigation. In addition, the explanation and questioning of symptoms increased the awareness about LUTS in individuals diagnosed with DMD and their caregivers.

### Limitations

The present study has several limitations. First, in this study, in which we investigated LUTS in children with DMD, we did not ask about all symptoms defined by the ICCS, but only the symptoms in the DVISS questionnaire. Symptoms of reduced voiding frequency, nocturia, hesitancy, weak stream, and post-micturition dribbling, which were not covered in the DVISS, were not assessed. However, the DVISS was used in this study because it is recommended by international associations and the literature. It is also an easy-to-use evaluation method. Another limitation is that some families may have low awareness of LUTS. Families may not see the symptoms as a problem because they accept the existing symptoms as normal. Some families are very resistant to the presence of these symptoms and may refuse even if they have symptoms. In addition, LUTS may be incorrectly reported by children. Such difficulties may have complicated the assessment of LUTS and influenced the results of symptom frequency. To minimize the effect of this situation on the results, the symptoms were explained to the families in detail with examples. In the sample, non-ambulatory and ambulatory groups coexist and there is no homogeneous distribution. Despite these limitations, our study is important to raise awareness among families and related health professionals.

The number of studies on LUTS in DMD is insufficient, and evaluation and treatment studies on the subject should be planned. Future studies should be planned to assess all LUTS in individuals with DMD. In particular, they should focus on individuals with DMD in the non-ambulatory stage. In addition, the frequency of LUTS and related factors can be studied in neuromuscular diseases other than DMD.

## Conclusion

Although LUTD is an overlooked condition, it is quite common in children with DMD. As a result of the presence of LUTS and LUTD, the participation of children with DMD in ADL, their level of independence, and their QoL are negatively affected. Despite its negative effects on children with DMD, urological evaluations are also neglected. Regular urological evaluations of children with DMD are important. Accordingly, awareness should be raised among all healthcare professionals working with patients with DMD.

### Supplementary Information

Below is the link to the electronic supplementary material.

Graphical abstract (PPTX 161 KB)

## Data Availability

Data available on request to the authors.

## Declarations

### Permission to reproduce material from other sources

No material from any other source was used. The tables, figures and photographs used are original, unpublished materials.

## References

[1] Bushby K, Finkel R, Birnkrant DJ, Case LE, Clemens PR, Cripe L et al (2010) Diagnosis and management of Duchenne muscular dystrophy, part 1: diagnosis, and pharmacological and psychosocial management. Lancet Neurol 9:77–93. 10.1016/S1474-4422(09)70271-6 19945913 10.1016/S1474-4422(09)70271-6

[2] Min YL, Bassel-Duby R, Olson EN (2019) CRISPR correction of Duchenne muscular dystrophy. Annu Rev Med 70:239–255. 10.1146/annurev-med-081117-010451 30379597 10.1146/annurev-med-081117-010451 PMC6415693

[3] Bhatt JM (2016) The epidemiology of neuromuscular diseases. Neurol Clin 34:999–1021. 10.1016/j.ncl.2016.06.017 27720006 10.1016/j.ncl.2016.06.017

[4] Morse CI, Higham K, Bostock EL, Jacques MF (2020) Urinary incontinence in men with Duchenne and Becker muscular dystrophy. PLoS One 15:e0233527. 10.1371/journal.pone.0233527 32469921 10.1371/journal.pone.0233527 PMC7259643

[5] van Wijk E, Messelink BJ, Heijnen L, de Groot IJ (2009) Prevalence and psychosocial impact of lower urinary tract symptoms in patients with Duchenne muscular dystrophy. Neuromuscul Disord 19:754–758. 10.1016/j.nmd.2009.07.009 19853790 10.1016/j.nmd.2009.07.009

[6] Brooke MH, Fenichel GM, Griggs RC, Mendell JR, Moxley R, Miller JP et al (1983) Clinical investigation in Duchenne dystrophy: 2. Determination of the “power” of therapeutic trials based on the natural history. Muscle Nerve 6:91–103. 10.1002/mus.880060204 6343858 10.1002/mus.880060204

[7] Santos ALYDS, Maciel FKDL, Fávero FM, Grossklauss LF, de Sá CDSC (2021) Trunk control and upper limb function of walking and non-walking Duchenne muscular dystrophy individuals. Dev Neurorehabil 24:435–441. 10.1080/17518423.2020.1869337 33412969 10.1080/17518423.2020.1869337

[8] Vignos PJ Jr, Spencer GE Jr, Archibald KC (1963) Management of progressive muscular dystrophy of childhood. JAMA 184:89–96. 10.1001/jama.1963.03700150043007 13997180 10.1001/jama.1963.03700150043007

[9] Akbal C, Genc Y, Burgu B, Ozden E, Tekgul S (2005) Dysfunctional voiding and incontinence scoring system: quantitative evaluation of incontinence symptoms in pediatric population. J Urol 173:969–973. 10.1097/01.ju.0000152183.91888.f6 15711352 10.1097/01.ju.0000152183.91888.f6

[10] Austin PF, Bauer SB, Bower W, Chase J, Franco I, Hoebeke P et al (2016) The standardization of terminology of lower urinary tract function in children and adolescents: Update report from the standardization committee of the International Children’s Continence Society. Neurourol Urodyn 35:471–481. 10.1002/nau.22751 25772695 10.1002/nau.22751

[11] Collin C, Wade DT, Davies S, Horne V (1988) The Barthel ADL Index: a reliability study. Int Disabil Stud 10:61–63. 10.3109/09638288809164103 3403500 10.3109/09638288809164103

[12] Mahoney FI, Barthel DW (1965) Functional evaluation: The Barthel Index: A simple index of independence useful in scoring improvement in the rehabilitation of the chronically ill. Md State Med J 14:61–65 14258950

[13] Varni JW, Seid M, Rode CA (1999) The PedsQL: measurement model for the pediatric quality of life inventory. Med Care 37:126–139. 10.1097/00005650-199902000-00003 10024117 10.1097/00005650-199902000-00003

[14] Iannaccone ST, Hynan LS, Morton A, Buchanan R, Limbers CA, Varni JW (2009) The PedsQL in pediatric patients with Spinal Muscular Atrophy: feasibility, reliability, and validity of the Pediatric Quality of Life Inventory Generic Core Scales and Neuromuscular Module. Neuromuscul Disord 19:805–812. 10.1016/j.nmd.2009.09.009 19846309 10.1016/j.nmd.2009.09.009 PMC2796341

[15] Napitupulu D, Abdillah L, Rahim R, Abdullah D, Setiawan M, Ahmar A et al (2018) Analysis of student satisfaction toward quality of service facility. Journal of Physics: Conference Series 954:012019. 10.1088/1742-6596/954/1/012019 10.1088/1742-6596/954/1/012019

[16] Bertrand LA, Askeland EJ, Mathews KD, Erickson BA, Cooper CS (2016) Prevalence and bother of patient-reported lower urinary tract symptoms in the muscular dystrophies. J Pediatr Urol 12:398.e1-e4. 10.1016/j.jpurol.2016.04.051 27567595 10.1016/j.jpurol.2016.04.051

[17] MacLeod M, Kelly R, Robb SA, Borzyskowski M (2003) Bladder dysfunction in Duchenne muscular dystrophy. Arch Dis Child 88:347–349. 10.1136/adc.88.4.347 12651768 10.1136/adc.88.4.347 PMC1719531

[18] Lionarons JM, de Groot IJM, Fock JM, Klinkenberg S, Vrijens DMJ, Vreugdenhil ACE et al (2021) Prevalence of bladder and bowel dysfunction in Duchenne muscular dystrophy using the childhood bladder and bowel dysfunction questionnaire. Life (Basel) 11:772. 10.3390/life11080772 34440515 10.3390/life11080772 PMC8399211

[19] Bendixen RM, Lott DJ, Senesac C, Mathur S, Vandenborne K (2014) Participation in daily life activities and its relationship to strength and functional measures in boys with Duchenne muscular dystrophy. Disabil Rehabil 36:1918–1923. 10.3109/09638288.2014.883444 24499260 10.3109/09638288.2014.883444 PMC4125555

[20] Palmer MH, Athanasopoulos A, Lee KS, Takeda M, Wyndaele JJ (2012) Sociocultural and environmental influences on bladder health. Int J Clin Pract 66:1132–1138. 10.1111/ijcp.12029 23163494 10.1111/ijcp.12029

[21] Uzark K, King E, Cripe L, Spicer R, Sage J, Kinnett K et al (2012) Health-related quality of life in children and adolescents with Duchenne muscular dystrophy. Pediatrics 130:e1559-1566. 10.1542/peds.2012-0858 23129083 10.1542/peds.2012-0858

[22] Birnkrant DJ, Bushby K, Bann CM, Apkon SD, Blackwell A, Brumbaugh D et al (2018) Diagnosis and management of Duchenne muscular dystrophy, part 1: diagnosis, and neuromuscular, rehabilitation, endocrine, and gastrointestinal and nutritional management. Lancet Neurol 17:251–267. 10.1016/S1474-4422(18)30024-3 29395989 10.1016/S1474-4422(18)30024-3 PMC5869704

[23] Iscan B, Ozkayın N (2020) Evaluation of health-related quality of life and affecting factors in child with enuresis. J Pediatr Urol 16:195.e1-e7. 10.1016/j.jpurol.2019.12.018 32008988 10.1016/j.jpurol.2019.12.018

[24] Carlton J, Powell PA; Project HERCULES Carer Group (2022) Measuring carer quality of life in Duchenne muscular dystrophy: a systematic review of the reliability and validity of self-report instruments using COSMIN. Health Qual Life Outcomes 20:57. 10.1186/s12955-022-01964-4 35366897 10.1186/s12955-022-01964-4 PMC8977045

