# Supplementary material for: The relationship between lower urinary system symptoms and the level of independence and quality of life in children with Duchenne muscular dystrophy
Source: Pediatr Nephrol. 2024 Jun 1;39(10):3005–12. doi: 10.1007/s00467-024-06419-0 (PMC11349853; doi:10.1007/s00467-024-06419-0)
Supplement: Supplementary file 1 — Graphical abstract (PPTX 161 KB) [file 467_2024_6419_MOESM1_ESM.pptx]

## Slide 1
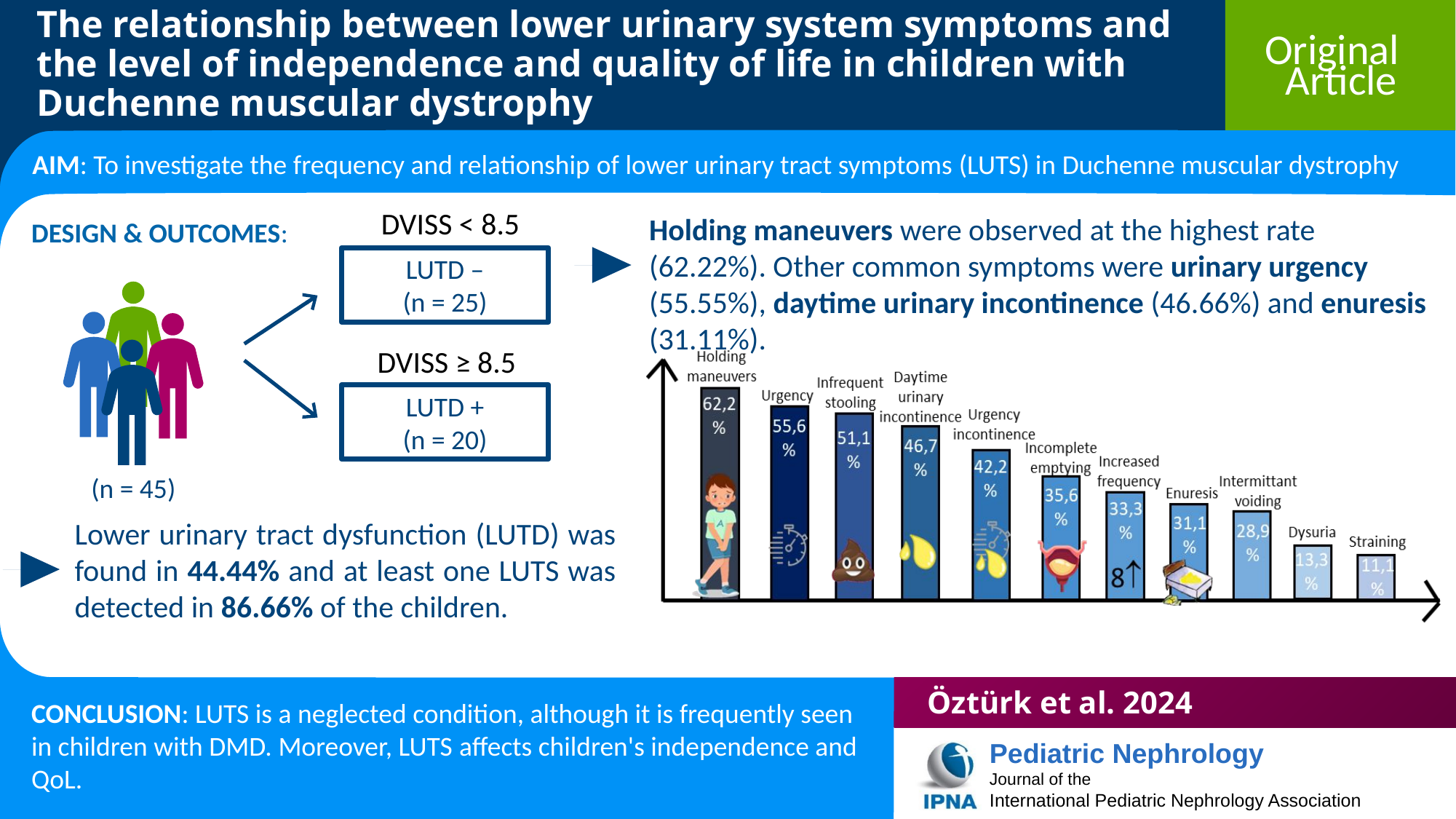

The relationship between lower urinary system symptoms and the level of independence and quality of life in children with Duchenne muscular dystrophy
AIM: To investigate the frequency and relationship of lower urinary tract symptoms (LUTS) in Duchenne muscular dystrophy
DVISS < 8.5
Holding maneuvers were observed at the highest rate (62.22%). Other common symptoms were urinary urgency (55.55%), daytime urinary incontinence (46.66%) and enuresis (31.11%).
DESIGN & OUTCOMES:
LUTD –
(n = 25)
DVISS ≥ 8.5
LUTD +
(n = 20)
(n = 45)
Lower urinary tract dysfunction (LUTD) was found in 44.44% and at least one LUTS was detected in 86.66% of the children.
Öztürk et al. 2024
CONCLUSION: LUTS is a neglected condition, although it is frequently seen in children with DMD. Moreover, LUTS affects children's independence and QoL.
